# Early learning difficulties, childhood stress, race, and risk of cognitive impairment among US adults over age 50: A cross‐sectional analysis

**DOI:** 10.1002/hsr2.1756

**Published:** 2023-12-12

**Authors:** Allan K. Nkwata, Jacqui Smith

**Affiliations:** ^1^ Institute for Social Research University of Michigan Ann Arbor Michigan USA

**Keywords:** childhood stressors, cognitive impairment, dementia, early learning problems, race

## Abstract

**Background and Aims:**

Most literature linking childhood factors to cognitive health outcomes has focused on educational attainment—defined as years of education attained. However, less has been studied about the other aspects of education, such as early learning problems, and stressful family environments. This study examined whether early learning problems and childhood stressors were associated with mid‐ and later life cognitive impairment among US adults, and if these associations varied by race.

**Methods:**

We conducted a cross‐sectional analysis using the Health and Retirement Study (HRS) along with respondents' early educational experiences from the 2015 to 2017 Life History Mail Survey (*N* = 9703). Early learning problems were defined as having any of the following: scholastic problems (reading, writing, mathematics), speaking/language issues, and sensorimotor issues‐ hearing, vision, speech, and motor‐coordination. Cognitive status was classified as three levels (normal, cognitively impaired not demented [CIND], and demented) using the HRS Langa−Weir algorithm. Multinomial logistic regression models using generalized logits, estimated relative risk ratios (RRRs), and 95% confidence intervals (CI) with adjustment for sociodemographic factors.

**Results:**

Having at least one early learning problem was associated with increased risk of later life cognitive impairment (RRR: 1.75, 95% CI: 1.34−2.29 for dementia, RRR: 1.42, 95% CI: 1.20−1.67 for CIND). Parental death before the age of 16 was associated with 17% higher risk of CIND in later life (RRR: 1.17, 95% CI: 1.01−1.34). However, learning problem‐related differences in risk of cognitive impairment were dependent on race (learning problems × race, *p* = 0.0001). In the demented group, Blacks were 2.7 times more likely to be demented (RRR: 2.66, 95% CI: 1.69−4.17) amongst older adults that experienced childhood learning problems.

**Conclusions:**

Early life exposures predicted risk of cognitive impairment. Policies and interventions that enhance diagnosis of early learning problems and improve childhood social contexts are needed to promote healthy cognitive aging amongst Americans, regardless of race.

## Key points

- Early life factors‐learning problems predicted risk of mid‐ and later life cognitive impairment.
- Contextual factors in childhood such as stressful school and family situations are independently important considerations for risk of cognitive impairment in mid‐ and late adulthood.
- Association between early life factors and risk of mid‐ and later life cognitive impairment varied by race.

## 1 INTRODUCTION

Among sociodemographic determinants of cognitive health, educational attainment is particularly important. Educational attainment has been associated with several beneficial effects across the life course that include enhanced reasoning skills, test‐taking abilities, verbal, and working memory—all of which translate to cognitive reserve, personal mastery, better health behaviors, income, and social network opportunities.^1^, ^2^, ^3^, ^4^ Education is also an important factor in determining level of impairment on screening tests for dementia^5^ as well as in‐depth neuropsychological measures. An individual's performance on cognitive tests is typically judged according to the deviation from average performance of others with similar age and level of education. However, most literature on education has generally focused on the amount of schooling one completes,^6^ but has yet to comprehensively examine other aspects of education, such as early learning problems or disorders.

Additionally, there is a shortage of literature on the long‐term consequences of early childhood learning difficulties on mid‐ and late life cognitive health outcomes. Prior research suggests that children with early learning problems may be deficient in numerical understanding and domain—functioning in language and spatial skills compared with their average achieving counterparts.^7^ Children with early learning problems may also be at risk for repeating a grade, maladjustment in social and emotional functioning, as well as dropping out in high‐school.^8^ For instance, in an earlier study that included 8−12 year old children assessed for childhood neurological symptoms, the authors found that about two‐thirds of students with learning disabilities dropped out of high school.^9^

Given the central role the brain plays in stress and adaptation, early learning problems can be viewed as stressors in childhood that will negatively impact the developing brain leading to poorer cognitive health across the life course.^10^ Furthermore, early childhood is a sensitive period for brain and body development, and chronic stress—whether severe or persistent, encountered in childhood can have devastating effects on later life brain health,^11^ contributing to the “long arm” of childhood disadvantage observed for various health outcomes.^12^, ^13^ Childhood chronic stressors may include child maltreatment and traumatic experiences related to family dysfunction, such as parental divorce, mental health, substance abuse problems, and incarceration among others.^14^ A compelling body of literature comes from studies of children raised in poverty or mistreated by their parents, who show heightened vulnerability to vascular disease, autoimmune disorders, and premature mortality.^15^, ^16^, ^17^ Several studies including the Adverse Childhood Experiences study, have shown that, among people exposed to psychosocial trauma in childhood, there are elevated rates of morbidity and mortality from chronic diseases of aging. For instance, one study that compared the medical outcomes of over 17,000 adults who did versus did not experience stressors like familial violence, abuse, and neglect as children found a greater likelihood of cardiovascular disease, autoimmune disorders, and premature mortality among those exposed to early childhood adversity.^18^

From a life course perspective, early childhood is a developmentally sensitive period to disruptions in family structure and context as it involves heightened susceptibility to external influences.^11^ Major disruptions in a family such as death of a parent, parental divorce, or separation, being unemployed for long periods can be stressful for children and may negatively impact them through life via numerous behavioral problems. Loss of a parent, especially early in childhood, breaks the bonds that were forming between parent and child, and has been associated with negative effects on the child's well‐being and an increased likelihood of or a greater vulnerability to long term adversity.^19^, ^20^ It is almost always followed by a decline in financial resources, social support, child support, poor self‐image, and lifestyle changes.^21^ Furthermore, parental death may hinder one's ability to form meaningful relationships through life,^16^, ^22^ leading to social isolation,^23^ and has also been associated with lower educational and income attainment.^24^, ^25^ With regard to separation, findings from the Fragile Families and Child Well‐being Study showed that high levels of household instability and complexity were important factors that shaped the experience of many low SES, never‐married single parents leading to poor outcomes in children.^26^ Research suggests that family financial hardship may affect child development via disrupted family processes such as marital distress and harsh parenting.^27^

Health disparities across social classes have long been recognized for various outcomes^28^ and these differences extend to children as well.^29^, ^30^ Prior research has established clear links between poverty and adversity in childhood and adult ill health.^31^ But poverty also increases the risk of childhood illness. For example, in one study, the authors found that sharp increases in parent‐reported child ill‐health followed increases in social disadvantage related to poverty, single parenthood, and lower educational attainment.^30^ Poverty also increases the risk of developmental delays, obesity, and asthma among children and makes chronic inflammation more likely.^32^

Similarly, geographic disparities in health outcomes across the United States have persisted for decades. For instance, stroke mortality rates differ markedly by region for US‐born Black and White folks. The Southeastern region is known for disproportionately high rates of stroke and cardiovascular disease morbidity and mortality especially amongst Blacks that were born or lived there.^33^ Evidence shows that these disparities are predicted by social or individual risk factors for example, lingering effects of segregation and racism, lower SES, lower educational quality and attainment, and higher unemployment rates amongst Blacks.^34^

Despite prior work linking childhood trauma to various health outcomes,^14^ the effects of childhood stressors such as death of a parent, family financial stress, and hindrances to school progress on later life cognitive functioning have generally been overlooked. Only a few studies have evaluated these associations. For instance, in a study conducted in Europe that assessed the association between early parental death and the risk of dementia in adult life, the authors found that experiencing parental death before age 16 was associated with an increased risk of dementia among older Europeans.^35^ Another study performed in Israel also found that parental death during childhood increased dementia risk in later life, however, on the contrary, parental death after age 18 was associated with decreased dementia risk.^36^ Similarly, findings from the Chinese Longitudinal Healthy Longevity Survey also showed that older men who experienced the death of a mother before age 16 reported higher odds of cognitive impairment than those who did not experience loss.^37^ The present study, therefore, aimed to examine the role of early childhood learning difficulties and childhood stressors on mid‐ and late‐life cognitive health in a large nationally representative sample of midlife and older adults. Beside a life course approach, this study is grounded in the cumulative inequality framework.^38^ This framework incorporates the cumulative burden of chronic stressors, aspects of household dysfunction, and life events and involves the interaction of different physiological and ecological systems. We hypothesized that (1) individuals with early learning problems will have poorer mid‐and later life cognitive health and (2) due to the impact that stress might have on the developing brain, individuals that experienced higher levels of childhood stress experiences will have poorer cognitive health in mid‐ and later life. We farther explored race as a potential modifier of the relationship between a history of early learning difficulties, childhood stressors, and cognitive impairment in our sample.

## 2 METHODS

### 2.1 Study population

This cross‐sectional analysis utilized panel interview data from the Health and Retirement Study (HRS) as well as information from respondents' early educational experiences collected in the 2015 and 2017 HRS Life History Mail Survey (LHMS)^39^ and cross‐wave aggregated panel child and family history.^40^ The HRS is conducted under the University of Michigan and sponsored by the National Institute of Aging and the Social Security Administration. Details of the design and implementation of the study have been widely described elsewhere.^41^ Briefly, the HRS began in 1992 and follows individuals every 2 years with detailed assessments of health outcomes, health expenditures, psychosocial, and lifestyle factors. The study has been approved by the University of Michigan Institutional Review Board. Our sample included 9703 participants who were at least 50 years old in the year 2016. We therefore, defined mid‐life to include adults who were aged 50−64, and late life to include individuals greater than 65 years old, consistent with aging literature.^42^ Reasons for study exclusion included age less than 50, no 2016 cognition data available, or deceased. Participants excluded were similar to those included with respect to sociodemographic factors.

### 2.2 Primary determinants: Early learning problems and childhood stressors

The primary exposures in this study were early learning problems, and childhood stress measures. Data on early learning experiences and childhood stress came from 2015 to 2017 LHMS^39^ and the aggregated child and family history data.^40^ Early learning problems included responses to the question: “In primary or elementary school, did any teachers, principals or psychologists tell you or your parents that you had a problem with learning any of the usual school subject, or speaking/language issues.” Subjects included reading, writing, math, and speaking/language problems. Additionally, we treated sensorimotor issues, such as problems with hearing, vision, speech, and motor‐coordination, as early learning problems. Sensorimotor issues may be linked to the input to the brain (i.e., impaired information to be processed)—or the “wiring” of the brain and could potentially contribute to learning difficulties.^43^, ^44^ For analytic purposes, early learning problems were defined as having any of the above eight problems. This was because the prevalence of individual learning problems was very low in our sample (data not shown).

We measured childhood stress with self‐reported responses to questions that capture stressful family situations encountered in childhood.^39^, ^40^ These relate to household financial difficulty, death of a parent, and having to repeat a year of school over again. We assessed financial difficulty from responses to the question: “Before age 16, was there a time of several months or more when your father had no job?” We measured death of a parent based on responses to the question: “Before you were age 16, did one or both of your biological or adoptive parents die?” We evaluated repeating school from responses to the question: “Before you were 18 years old, did you have to do a year of school over again?” For analytic purposes, we treated these questions as categorical (yes/no) variables and examined them separately.

### 2.3 Outcome measure: Cognitive impairment

The HRS assesses cognitive function using the modified Telephone Interview for Cognitive Status (TICSm), a validated cognitive screening tool that has been widely used in population‐based studies to evaluate cognition.^45^ Briefly, individual cognitive functioning measures include immediate and delayed word recall, the serial 7 s test, questions on counting backwards, naming tasks such as date naming, and vocabulary questions. We utilized a summary index‐ a 27‐point scale (range 0−27) from the above items, to determine total cognitive functioning.^46^ We classified cognitive status as three different levels; normal for a total cognitive functioning score of 12−27, cognitively impaired but not demented (CIND) for a score of 7−11, and demented for a score of 0−6, consistent with Langa−Weir's algorithm.^47^

### 2.4 Covariates

Other factors that we controlled for included, age as a continuous variable, sex, race, years of education, marital status, and census region of birth, and adult chronic conditions. Respondents were asked if they had ever been diagnosed with any of the following chronic conditions: hypertension, diabetes, cancer, chronic lung disease, heart attack, coronary heart disease, congestive heart failure, stroke, arthritis, and emotional, nervous, or psychiatric problems. However, for analytic purposes, we created a comorbidity index as a categorical variable (yes/no) to include three chronic conditions (heart disease, stroke, and diabetes) that are established risk factors for dementia.^48^ This variable tracked participants with at least one of the above three chronic conditions.

### 2.5 Statistical analysis

We analyzed early learning problems, childhood stress experiences, and race as predictors of later life cognitive impairment. First, we performed descriptive analyses to evaluate the distribution of sociodemographic factors and childhood stress measures by early learning problems. We then evaluated bivariate associations to determine crude associations of early learning problems, and sociodemographic factors with later life cognitive impairment. Since both early learning problems and childhood stressors were analyzed as categorical variables, we used *χ* ^2^ tests to evaluate differences in proportions. Factors with a *p*‐value ≤ 0.2 were further evaluated in multivariable models as candidate confounders. Next, we ran a series of multinomial logistic regression models with generalized logits, having individuals with normal cognitive function as the reference group, to estimate relative risk ratios (RRRs) and 95% confidence intervals (CIs) with adjustment for potential confounders such as age, sex, race, marital status, years of education, chronic conditions, and region of birth. These logit models evaluated cognition on two different levels: CIND or demented versus the normal (referent group). In the first set of models, we considered early learning problems as potential predictors of mid‐ and later life cognitive impairment, controlling for sociodemographic factors. A second set of models included childhood stressors as potential predictors of mid‐ and later life cognitive impairment, controlling for sociodemographic factors as well. A third set of models included early learning problems and childhood stress measures. Additionally, separate models evaluated interaction between early learning problems, childhood stress and race. Since these moderation analyses were exploratory and expected to be underpowered, interaction terms with *p*‐values < 0.10 were considered potentially important,^49^ and stratum specific results presented where potential interactions were indicated. All analyses were performed with SAS software, version 9.4 (SAS Institute).

## 3 RESULTS

Included in the sample were 9703 respondents interviewed in the 2016 HRS wave, with age range 50−101 years, White (73%), women (61%), and college education or more (33%). 14.8% were CIND, while 4.7% were demented. Individuals with early learning problems were on average 3 years younger than those without (66.9 vs. 69.9 years, respectively). Detailed sample characteristics of the study participants by learning problem status are listed in Table 1.

**Table 1:** Demographic characteristics of the 2016 wave of HRS overall and by learning problem status.

Characteristics	Overall (*N* = 9703)	Any (*N* = 1897)	None (*N* = 7806)	*p* Value
Sociodemographic	*N* (%)	*N* (%)	*N* (%)	
Age in years: mean (SD)	69.31 (9.68)	66.86 (9.09)	69.90 (9.72)	
Sex: Women	5865 (60.4)	1118 (58.9)	4747 (60.8)	0.1337
Race: White	7220 (74.4)	1374 (72.4)	5846 (74.9)	0.0866
Black	1783 (18.4)	376 (19.8)	1407 (18.0)	
Other race	699 (7.2)	147 (7.7)	552 (7.1)	
Region of birth: Northeast	1705 (17.6)	385 (20.3)	1320 (16.9)	<0.0001
Midwest	2626 (27.1)	507 (26.7)	2119 (27.2)	
South	3012 (31.1)	535 (28.2)	2477 (31.7)	
West	1012 (10.4)	250 (13.2)	764 (9.8)	
Foreign	1342 (13.8)	220 (11.6)	1122 (14.4)	
Marital status: Married	5725 (59.1)	1062 (56.1)	4663 (59.9)	<0.0001
Separated/divorced	1584 (16.4)	370 (19.5)	1214 (15.6)	
Widowed	1858 (19.2)	315 (16.6)	1543 (19.8)	
Never married	510 (5.3)	146 (7.7)	364 (4.7)	
Education: Less than high school	1878 (19.3)	398 (21.0)	1480 (19.0)	0.0464
High school graduate	4585 (47.2)	853 (44.9)	3732 (47.8)	
Some college and above	3240 (33.4)	646 (34.0)	2594 (33.2)	
Comorbid heart disease, diabetes, or stroke	4476 (46.1)	906 (47.8)	3570 (45.7)	0.1124
Childhood stressors				
Had to repeat a year of school over again	1290 (14.9)	412 (25.0)	878 (12.5)	<0.0001
Parent died before age 16	2065 (22.1)	414 (22.7)	1651 (21.9)	0.4379
Father unemployed in childhood	1842 (19.2)	391 (20.7)	1451 (18.8)	0.0545
Cognitive functioning status				
Normal	7803 (80.4)	1475 (77.7)	6328 (81.1)	0.0049
Cognitively impaired but not demented (CIND)	1440 (14.8)	321 (16.9)	1119 (14.3)	
Demented	460 (4.7)	101 (5.3)	359 (4.6)	

Abbreviation: HRS, health and retirement study.

### 3.1 Early learning problems are associated with later life cognitive impairment, disparities persist according to race

Having at least one early learning problem was associated with increased risk of later‐life cognitive impairment (mild/CIND) and dementia, after adjusting for age, sex, race, socioeconomic status, chronic conditions, and region of birth (Model 1 and Table 2). First, comparing the demented versus the normal (referent) group, the relative risk of being demented versus being normally cognitive in later life increased by 73% (RRR: 1.73, 95% CI: 1.34−2.22) for individuals that experienced any early learning problems versus none (Table 2). Comparing the CIND to the normal (referent) group, the relative risk of CIND increased by 52% (RRR: 1.52, 95% CI: 1.31−1.74) for individuals that experienced any early learning problems versus none. These associations remained strong with farther adjustment for childhood stressors (Model 3 and Table 2).

**Table 2:** Early learning problems, childhood stressors, and race in relation to risk for cognitive impairment among older American adults enrolled in the HRS wave of 2016.

Characteristic	OR (95% confidence interval)		*p* Value
**Model 1**	**Demented**	**CIND**	
**Early learning difficulties**			
Any versus none	**1.73 (1.34−2.22)**	**1.52 (1.31−1.74)**	<0.0001
**Model 2 (with childhood stressors)**			
**Had to repeat a year of school over again**			
Yes versus no	**1.34 (1.02−1.77)**	**1.34 (1.13−1.59)**	0.0012
**Death of a parent**			
Yes versus no	1.25 (0.99**−**1.57)	**1.18 (1.03−1.36)**	0.025
**Father unemployed in childhood**			
Yes versus no	1.20 (0.94**−**1.53)	**1.19 (1.02−1.38)**	0.046
**Model 3 (with childhood stressors and early learning difficulties)**			
**Early learning difficulties**			
Any versus none	**1.75 (1.34−2.29)**	**1.42 (1.20−1.67)**	<0.0001
**Had to repeat a year of school over again**			
Yes versus no	1.22 (0.92**−**1.62)	**1.27 (1.07−1.51)**	0.0162
**Death of a parent**			
Yes versus no	1.24 (0.99**−**1.56)	**1.17 (1.01−1.34)**	0.038
**Father unemployed in childhood**			
Yes versus no	1.18 (0.93**−**1.50)	**1.19 (1.03−1.38)**	0.047
**Race**			<0.0001
Blacks versus Whites	**2.68 (2.01−3.55)**	**2.83 (2.38−3.38)**	
Other Race versus Whites	**2.40 (1.57−3.67)**	**1.97 (1.51−2.56)**	

*Note*: Model 1 includes early learning problems and adjusts for age, race, sex, marital status, years of education, region of birth, and chronic conditions; Model 2 includes childhood stressors and adjusts for age, race, sex, marital status, years of education, region of birth, and chronic conditions; Model 3 includes early learning problems, childhood stressors and adjusts for age, race, sex, years of education, region of birth, and childhood stressors. Bold indicates statistically significant results.

Abbreviations: CIND, cognitively impaired not demented; HRS, health and retirement study.

**Figure 1:**
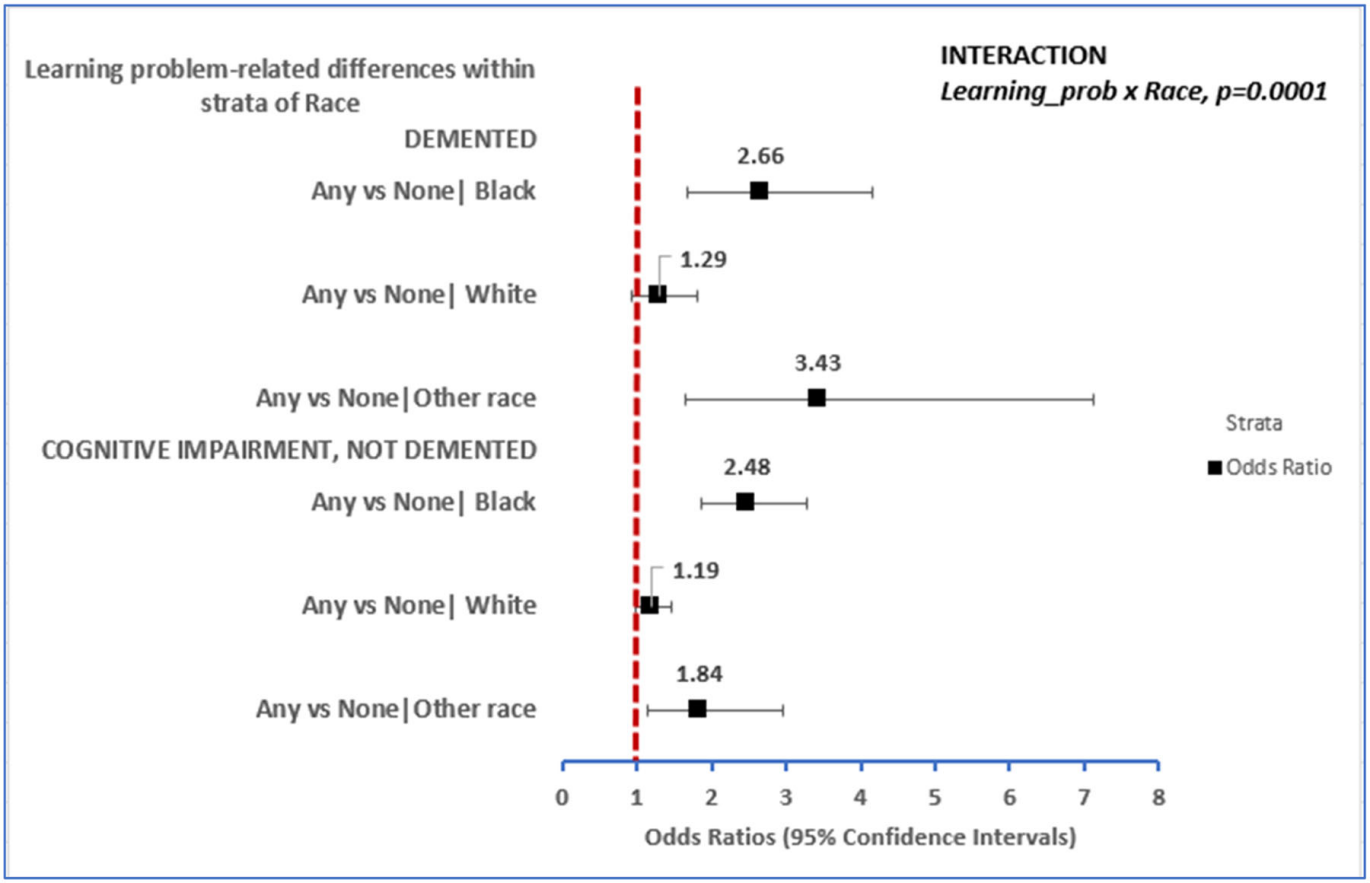
Learning problem‐related differences in risk of cognitive impairment varied within strata of race among older American adults enrolled in the HRS wave of 2016. HRS, health and retirement study.

However, learning problem‐related differences in risk of cognitive impairment were dependent on race (learning problems × race, *p* = 0.0001, Figure 1). Amongst Blacks and other race, experiencing early learning problems was associated with risk of cognitive impairment when demented or CIND were compared with the normal group. For instance, among Blacks in the demented group, the expected risk of being demented was 2.7 times higher (RRR: 2.66, 95% CI: 1.69−4.17) for individuals that experienced any early childhood learning problems versus none. Similarly, amongst Blacks in the CIND group, the expected risk of being cognitively impaired was 2.5 times higher (RRR: 2.48, 95% CI: 1.87−3.27) for those that experienced any early childhood learning problems. Amongst other race individuals (demented group), the expected risk of being demented was 3.4 times higher (RRR: 3.43, 95% CI: 1.66−7.11) for individuals that experienced any early childhood learning problems. However, amongst Other race individuals in the CIND group, the expected risk of being cognitively impaired was 1.8 times higher (RRR: 1.84, 95% CI: 1.14−2.96) for those that experienced any early childhood learning problems. On the contrary, amongst Whites the association between those with early learning problems versus none on later life cognitive impairment was not statistically significant (Figure 1).

With respect to the interactions between the domains of childhood stressors analyzed and race, these did not yield statistically significant findings (see Supporting Information S1: Table 1).

### 3.2 Childhood stressors associated with late life cognitive impairment

Death of a biological or adoptive parent before the age of 16 was associated with 18% higher risk of late life cognitive impairment (RRR: 1.18, 95% CI: 1.03−1.36, Model 2, Table 2). However, the association was not statistically significant among demented individuals (RRR: 1.25, 95% CI: 0.99−1.57). Having to repeat a year of school over again before age 18 was associated with 34% elevated risk of both dementia (RRR: 1.34, 95% CI: 1.02−1.77), and cognitive impairment in later life (RRR: 1.34, 95% CI: 1.13−1.59). Having a father unemployed for several months during childhood was not significantly associated with dementia risk amongst these older adults (RRR: 1.18, 95% CI: 0.93−1.50). However, among CIND adults, having a father unemployed for several months during childhood was associated with 19% increased risk of cognitive impairment in mid and later life (RRR: 1.19, 95% CI: 1.03−1.38, Model 2, Table 2). Worth noting, the expected risk of each of the childhood stressors assessed was somewhat attenuated with the adjustment of learning problems (Model 3 and Table 2).

## 4 DISCUSSION

In this study of 9703 older Americans, early learning problems in childhood were associated with increased risk of cognitive impairment in mid‐ and later life. Our findings are in line with our hypothesis that early learning problems would be associated with increased risk of cognitive impairment in mid‐ and later life. Our findings also corroborate those from a study that examined how early educational experiences—school context, content and academic ability, impacted cognitive functioning, and decline in later life, which found that early educational experiences were associated with late life cognitive decline, but only in the presence of a learning problem.^50^ While we focused on early learning problems together with household childhood stressors, experiencing early learning problems can limit a person's ability to pursue educational opportunities across life.^9^ Additionally, other studies have consistently identified a lower education status as a risk factor for cognitive impairment and dementia in older adults.^2^, ^51^ However, the present study extends the literature by evaluating early learning problems and contextual or social factors (childhood stressors) that may be associated with later life cognitive impairment, thereby contributing to the “long arm” of childhood adversity on adult health outcomes.^13^ Our findings also suggest that early‐life disadvantage if not alleviated, may result in cumulative inequality overtime,^38^ placing individuals at risk for a variety of later life adverse health outcomes for example, cancers, multiple chronic conditions, poor physical functioning, and lower self‐rated health.^52^, ^53^ Furthermore, we found novel empirical evidence that learning problem‐related differences in risk of cognitive impairment varied by race. We observed statistically significant associations between learning problems and cognitive impairment only amongst Black and other race Americans suggesting that issues leading to differential access to healthcare in childhood, for example, lower SES, lack of health insurance, and unemployment amongst minority race Americans may be drivers of these associations.

Data from this study farther suggests that stressful family contexts encountered during childhood for example, death of a biological or adoptive parent, having to repeat a year of school over again before the age of 18, and situations where a father was unemployed for long periods over 6 months, predicted later life cognitive impairment. From a life course perspective, the above contexts represent household disruptions that can activate lifelong patterns of psychological distress, that may increase the risk of later life cognitive impairment. Regarding parental death, the increase in life expectancies observed over several decades suggests that parental death tends to happen in mid to late life for most Americans. However, when loss of a parent happens in childhood or adolescence, this trauma may lead to subsequent negative patterns such as depression, health damaging behaviors, morbidity, and mortality in adulthood.^54^ Our findings that experiencing early parental death is a risk factor for later life cognitive impairment corroborate those from a few other studies conducted outside the US that examined the association between the early life parental death and later life cognitive health.^35^, ^36^, ^37^ A more recent study using HRS data evaluated cognitive impairment in relation to parental death from childhood through early adulthood, midlife, and later adulthood. The authors found that both exposure and timing of parental death increased the risk of cognitive impairment in later life and that these associations varied by gender.^55^

Similarly, our findings on father's unemployment and having to repeat a year of school generally echo those from other studies that assessed the long‐term impact of childhood stressors such as trauma and poor childhood SES on late life cognitive health.^56^, ^57^, ^58^ Childhood trauma resulting from socioeconomic strain has been linked to negative health impacts such as poor physical and mental health, behavioral problems, poor academic achievement, and depression across the life course.^59^, ^60^

While research has shown that early learning problems may persist lifelong, their effects may be reduced if adequate treatment/interventions are provided during childhood.^8^ Findings from the Fragile families study suggest that when fathers are empowered and are present in families, this reduces the risk of externalizing behaviors in children thus playing a protective role against the harmful effects of stressors.^61^ Important to note, the literature examining how early learning experiences impact late life cognitive function remains sparse. This is perhaps because hardly any studies collected reliable historical individual data on early learning problems and contexts in the past that could match the experiences of the birth cohorts we have in our study.

Strengths of the study include the fact that it sheds more light on the relationship between early education experiences and later life cognitive health, with a focus on early learning problems. We employed a life‐course approach to test direct associations between early learning problems and cognitive impairment. Additionally, we examined multiple indicators of childhood stress to see if they were associated with late‐life cognitive decline. We also evaluated two classifications of cognitive impairment—CIND (suspected dementia) and dementia. CIND includes individuals whose cognitive functioning scores fall below normal but who do not meet dementia criteria.^47^ Limitations of the study include its cross‐sectional design which limits causal inference due to potential for residual confounding by unmeasured factors and inability to infer temporal sequence. Also, self‐reported assessments of early learning problems and dementia diagnosis were used, allowing for potential information bias and recall bias despite the thorough efforts the HRS made to collect data in a standardized method.

## 5 CONCLUSIONS

This study provided further empirical evidence that early learning problems, childhood stressors, and race are important social determinants of cognitive impairment in a diverse sample of older US adults. This research suggests the need for policies/interventions that enhance quicker diagnosis of early learning problems, decrease early childhood stress and opportunities that enhance social equity to promote healthy cognitive aging amongst older Americans. Hence, early diagnosis of learning problems and timely interventions could mitigate the risk of cognitive impairment in later life.

## AUTHOR CONTRIBUTIONS

**Allan Nkwata**: Conceptualization, formal analysis, methodology, writing—original draft, writing—review and editing. **Jacqui Smith**: Conceptualization, funding acquisition, methodology, resources, writing—review and editing. All authors have read and approved the final version of the manuscript.

## CONFLICT OF INTEREST STATEMENT

The authors declare no conflict of interest.

## TRANSPARENCY STATEMENT

The lead author Allan K. Nkwata affirms that this manuscript is an honest, accurate, and transparent account of the study being reported; that no important aspects of the study have been omitted; and that any discrepancies from the study as planned (and, if relevant, registered) have been explained.

## Supporting information

Supporting information.

## Data Availability

[Allan Nkwata] had full access to all of the data in this study and takes complete responsibility for the integrity of the data and the accuracy of the data analysis. Publicly available data sets were analyzed in this study. This data can be found here: https://hrs.isr.umich.edu/data-products/access-to-public-data.
