# Supplementary material for: Early learning difficulties, childhood stress, race, and risk of cognitive impairment among US adults over age 50: A cross‐sectional analysis
Source: Health Sci Rep. 2023 Dec 12;6(12):e1756. doi: 10.1002/hsr2.1756 (PMC10716572; doi:10.1002/hsr2.1756)
Supplement: Supplementary file 1 — Supporting information. [file HSR2-6-e1756-s001.docx]

**Supplementary Tables**

Supplementary Table 1: Learning problem-related and childhood stress-related differences within strata of race in the 2016 HRS sample.

|  | **DEMENTED** | **CIND** |  |
| --- | --- | --- | --- |
| **Learning problem-related differences varied within strata of race** | **OR (95% Confidence Interval)** | | **Interaction p-value** |
| Amongst Blacks - Presence of learning problems: Yes vs No | **2.66 (1.69, 4.17)** | **2.48 (1.87, 3.27)** | **0.0001** |
| Amongst Whites-Presence of learning problems: Yes vs No | 1.29 (0.93, 1.81) | 1.19 (0.98, 1.45) |  |
| Amongst Other race- Presence of learning problems: Yes vs No | **3.43 (1.66, 7.11)** | **1.84 (1.14, 2.96)** |  |
| **Father's unemployment-related differences within strata of race** |  |  | 0.7918 |
| Amongst Blacks-Father unemployed: Yes vs No | 1.37 (0.85, 2.21) | 1.07 (0.78, 1.46) |  |
| Amongst Whites-Father unemployed: Yes vs No | 1.10 (0.48, 2.56) | **1.24 (1.04, 1.48)** |  |
| Amongst Other race-Father unemployed: Yes vs No | 1.11 (0.82, 1.49) | 1.15 (0.71, 1.87) |  |
| **Parental death-related differences within strata of race** |  |  | 0.872 |
| Amongst Blacks-Parent died: Yes vs No | 1.13 (0.75, 1.71) | 1.11 (0.86, 1.44) |  |
| Amongst Whites-Parent died: Yes vs No | **1.36 (1.01, 1.83)** | **1.23 (1.02, 1.47)** |  |
| Amongst Other race-Parent died: Yes vs No | 0.97 (0.48, 1.98) | 1.11 (0.72, 1.71) |  |
| **School-related differences within strata of race** |  |  | 0.4241 |
| Amongst Blacks-Repeated a year of school: Yes vs No | 1.44 (0.85, 2.44) | 1.04 (0.74, 1.47) |  |
| Amongst Whites-Repeated a year of school: Yes vs No | 1.12 (0.79, 1.58) | **1.40 (1.14, 1.71)** |  |
| Amongst Other Race-Repeated a year of school: Yes vs No | 1.33 (0.53, 3.37) | 1.13 (0.64, 2.01) |  |
